# Not all maggots are created equal; not all maggots are therapeutic

**DOI:** 10.1002/ccr3.9163

**Published:** 2024-07-17

**Authors:** Ronald A. Sherman, Albert Nguyen, Vahab Dastpak, Alicia Fonseca‐Muñoz, Naser Parizad, Mansour Siavash, Mustapha Ahmed Yusuf

**Affiliations:** ^1^ BioTherapeutics, Education and Research (BTER) Foundation Irvine California USA; ^2^ Faculty Member of Joundishapour Medical Science University of Ahwaz Health Technology Park and Pharmaceutical Technology Development Center Ahvaz Khuzestan Province Iran; ^3^ Facultad de Sistemas Biológicos e Innovación Tecnológica Universidad Autónoma “Benito Juarez” de Oaxaca Oaxaca Mexico; ^4^ Patient Safety Research Center Clinical Research Institute, Urmia University of Medical Sciences Urmia Iran; ^5^ Isfahan Endocrine and Metabolism research center Isfahan University of Medical Sciences Isfahan Iran; ^6^ Department of Medical Microbiology and Parasitology/Department of Microbiology Bayero University, Kano/Aminu Kano Teaching Hospital Kano Nigeria

## Abstract

Maggots in a wound (“myiasis”) cannot be considered maggot therapy (therapeutic myiasis) unless, at a minimum, the species is known to be safe and effective, and the maggots have been properly disinfected. Documenting treatment details is critical and allows us to determine the cause of problems, if they arise.

*Dear Editor,*

We read, with interest, the recent publication by Babazadeh et al., entitled: *Mistreatment with maggot therapy in diabetic foot ulcer causing an amputation*.^1^ The key point of the article is important enough to repeat here: “… if we do not pay attention to the indications and contraindications of [maggot therapy], there might be a failure in the treatment.” Indeed, a clinician cannot become competent in maggot therapy^2^ or any other skill unless they understand both the benefits and the risks associated with it. Careful review of that article reveals several other important lessons, too; and since those lessons were not specifically discussed in the publication, we believe they need to be discussed here.

First and foremost, one must always remember that maggot infestations should not be confused with maggot therapy. While maggot debridement therapy (MDT) is a controlled, therapeutic form of myiasis (maggot infestation on a live vertebrate host), not all cases of myiasis are legitimately called “maggot therapy.” This is because not all maggots (fly larvae) are beneficial; some can be quite destructive. Safe and effective therapy has resulted from using germ‐free *Lucilia* (*Phaenicia*) *sericata*, *L. cuprina*, *L. illustris*, *L. caesar, Phormia regina*, *Protophormia terraenovae, Calliphora vicina, Chrysomya rufifacies, Wohlfahrtia nuba*, and even *Musca domestica*.^3^ But even these can cause serious harm if not adequately disinfected.^4^ Applying nontherapeutic species can result in dire consequences^5^, ^6^ and should not be called “maggot therapy,” regardless of the intent. The method of application (larval age, dosage, type of dressings, and duration of treatment) and pre‐ and postprocedure wound care all can impact the clinical outcome of therapy, and therefore must be documented. For example, younger larvae debride slower and therefore may need more time on the wound; older larvae are larger and debride a wound faster but they can be felt more easily by patients with intact sensation and are more likely to cause pain; maggot containment dressings are easier to manage than maggots freely roaming around the wound bed, but the containment dressings restrict larval access to the wound and therefore commonly require longer treatment times (4 days vs. 2 days) and more treatments to reach complete debridement.^7^ In the report by Babazadeh and colleagues, no details were provided concerning the species, source, or disinfection methods used to ensure that the maggots were of medical grade. No information was presented about the dressing application (type and duration of dressings), or the training of the therapist (perhaps a clinician; perhaps a family member). This lack of information leaves us questioning whether or not the patient actually received a proper course of maggot therapy at all.

Let us also remember that—outside of the fact that he “attended a fungal specialist” and had “just one session of [maggot] therapy”—the patient's activities and wound care are totally unknown for the 2 months immediately preceding his return to the surgical team, when he required the amputation. Therefore, even if he did receive a proper 2–4 day course of maggot therapy, we cannot know for certain whether the deterioration of the wound really resulted from this single cycle of MDT or rather from any other treatments that he did or did not receive during the remaining 56+ days that he was out of the authors' care.

Our second teaching point is that the standard practice for photographically documenting the benefits or hazards of wound care treatments should be to include, at a minimum, both a pretreatment photograph and a posttreatment photograph. In this “Case Image” of “mistreatment with maggot therapy,” purportedly “causing an amputation,” the editor and reviewers accepted a single posttreatment wound image, taken immediately prior to amputation, as evidence that maggot therapy was so deleterious to the wound that amputation was now required. The lack of detailed written documentation would normally be acceptable for a photographic case report. But in this case, there were neither photographs nor text sufficient to detail the alleged damage caused by maggot therapy. As readers, we cannot comprehend the deleterious effects that the maggots may have had, because we do not know how serious the wound was before the maggots were even applied. Indeed, most clinicians we know do not even consider using maggot therapy until—and unless—the wound is already limb‐threatening.

Our third and final point is that much has been learned about the risks^8^, ^9^, ^10^, ^11^, ^12^, ^13^ and utility^14^, ^15^, ^16^, ^17^, ^18^, ^19^, ^20^ of maggot therapy over the past three decades. Many of the previously espoused absolute contraindications are now only warnings, or relative contraindications. Babazadeh et al. list a number of contraindications: “*Pseudomonas* *aeruginosa* infection and tendon exposure,” “dry wounds, open wounds in the abdominal cavity, pyoderma gangrenosum in patients with immunosuppressive therapy, and septic, arthritis wounds….” They point to two of these contraindications as being the reasons that maggot therapy was inappropriately applied to their patient's wounds: “In this case, *Pseudomonas* *aeruginosa* infection and tendon exposure are the reasons that maggot therapy must be avoided.” Given the lack of clinical data regarding this patient's wound before and after the application of maggots, we cannot comfortably attribute the wound progression and amputation in this case to maggot therapy. Most potential hazards associated with maggot therapy are preventable, as long as adequate preparations are made and precautions taken. There are certainly risks with maggot therapy, as with all interventions; but many clinicians have demonstrated successful outcomes treating *P. aeruginosa* infections,^21^ exposed tendons,^22^ eschar,^14^ open abdominal wounds,^14^ pyoderma gangrenosum,^23^, ^24^, ^25^ infected joints and joint prostheses,^22^, ^26^, ^27^ thoracotomy wounds,^14^ ischemic wounds,^28^, ^29^, ^30^, ^31^, ^32^ and a whole host of other problematic wounds that no one would have dreamed of treating 20 years ago. That is not to say that MDT is always successful; it is not. But failure to successfully treat a severe wound or prevent amputation is neither an adverse event nor a contraindication; it is simply a failure.

In summary, Babazadeh and colleagues provided us all the opportunity for several lessons in maggot therapy. They articulated the need to recognize the risks and limitations of our treatments and interventions. We enumerated three additional lessons in this letter. Readers of Babazadeh's paper have a good grasp of the catastrophic nature of their patient's situation, though many important details are missing: A man with diabetes and a history of cigarette smoking developed ulcers on his legs and feet. At some point after the third week and before the 12th week, he had some type of maggots placed on one or more wounds for maybe 2, 3, or 4 days. Nevertheless, he required amputation after about 3 months. Babazadeh and colleagues suggest that these maggots were medical grade maggots, applied therapeutically by a reasonably experienced person. The maggots—and the medical procedure known as maggot therapy—now stand accused of the crime of causing amputation, but convincing evidence is completely lacking. We, as jurors, vote for acquittal.

## AUTHOR CONTRIBUTIONS

**Ronald A. Sherman:** Conceptualization; investigation; methodology; project administration; supervision; writing – original draft. **Albert Nguyen:** Data curation; investigation; writing – review and editing. **Vahab Dastpak:** Investigation; writing – review and editing. **Alicia Fonseca‐Muñoz:** Investigation; writing – review and editing. **Naser Parizad:** Investigation; writing – review and editing. **Mansour Siavash:** Investigation; writing – review and editing. **Mustapha Ahmed Yusuf:** Investigation; writing – review and editing.

## FUNDING INFORMATION

No funding was received in support of this work.

## CONFLICT OF INTEREST STATEMENT

None of the authors claim any financial or personal relationship that could inappropriately influence or bias the content of this paper.

## ETHICS STATEMENT

The authors confirm that the approval of an institutional review board or patient consent was not required for this work. We confirm that we have read the Journal's position on issues involved in ethical publication and affirm that this work is consistent with those guidelines.

## CONSENT

Written informed consent was obtained from the patient to publish this report in accordance with the journal's patient consent policy.

## Data Availability

Data available upon request from the authors.
